# A Novel Hyaluronic Acid Filling Technique for Restoring Volume of the Labia Majora

**DOI:** 10.7759/cureus.45728

**Published:** 2023-09-21

**Authors:** Flavia Tarabini, Laila Rozemberg, Gisele Zapata-Sudo, Andre Braz

**Affiliations:** 1 Obstetrics and Gynecology, Federal University of Rio de Janeiro, Rio de Janeiro, BRA; 2 Dermatology, Clínica Ginecológica, Rio de Janeiro, BRA; 3 Anesthesiology, Federal University of Rio de Janeiro, Rio de Janeiro, BRA; 4 Dermatology, Clínica Dermatológica, Rio de Janeiro, BRA

**Keywords:** deflated labia majora, non invasive aesthetics, gynecological aesthetics, vulvar atrophy, hyaluronic fillers

## Abstract

Vulvar rejuvenation, which includes both functional and aesthetic aspects, has received a lot of attention in recent years. Despite the fact that surgical interventions have proven to be effective, the development of minimally invasive techniques for restoring volume and tissue function remains a top priority. This case study describes a novel method for vulvar volumization and collagen stimulation of the labia majora using a hyaluronic acid filling technique. The procedure begins with a meticulous assessment of each patient's anatomical characteristics and specific concerns, followed by hyaluronic acid retroinjections using a microcannula. The current article describes the use of this technique on a single patient and emphasizes its potential benefits in addressing various vulvar concerns, with a focus on minimal downtime and high patient satisfaction. The case report adds to the ongoing search for optimal vulvar rejuvenation strategies by providing valuable insights into the efficacy and utility of this novel approach.

## Introduction

The rejuvenation and treatment of functional and aesthetic vulva issues have grown in importance as women seek to address changes in genital tissues caused by factors such as aging, hormonal fluctuations, and childbirth. Modifications in the genital tissue can also be seen in athletes, women who have lost a lot of weight, or those who have anatomical or hypoplastic variations. In this context, symptoms such as decreased tone in the labia majora, decreased vaginal lubrication, decreased erotic sensation, vaginal laxity, mucosal fissures, and discomfort during sexual intercourse are associated with genital tissue modifications [1]. As a result, surgical and non-surgical interventions to alleviate these conditions have emerged, promoting improved quality of life and aesthetic solutions [2]. While genital surgical procedures have been extensively described, there is a paucity of literature on minimally invasive techniques for restoring volume and tissue function [3-5].

In response to this gap, we created an innovative technique that uses hyaluronic acid (HA) to restore volume and tone to the labia majora, specifically targeting the complaint of labia majora atrophy, which affects women at various stages of life. The goal of this article is to provide a detailed description of our technique, which involves volumizing and stimulating collagen in the labia majora with an 18-G, 50-mm-long microcannula. We aim to provide a minimally invasive and effective solution to address women's genital tissue issues and improve patient satisfaction by carefully assessing each patient's anatomical characteristics in the vulva tissue, individual concerns, and possible symptoms. This article will introduce this innovative technique, providing valuable insights for the development of optimal vulvar rejuvenation strategies.

## Case presentation

Technique explanation

The technique used in this patient involves the use of cross-linked hyaluronic acid, which has the ability to bind to water molecules and add volume and enhance tissue. Furthermore, HA has the ability to stimulate collagen production, which improves tissue quality. 

The examination is performed in an illuminated environment with the patient in a lithotomy position to determine the best location for volume restoration. This allows for the evaluation of vulvar morphology as well as the estimation of skin laxity. The treated anatomical region extends inferoposteriorly from the mons pubis to the perineum. 

**Figure 1:**
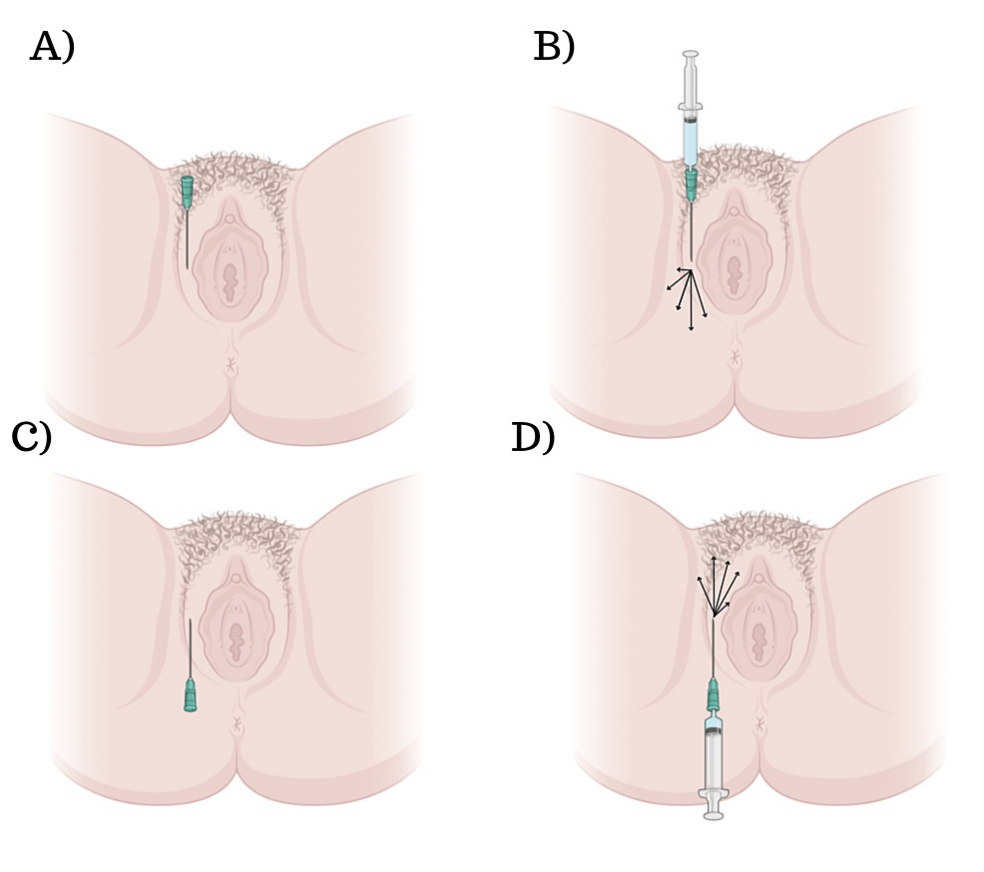
A schematic representation of our technique. A) Creation of the orifice using a needle, followed by insertion of the microcannula. B) Attachment of the syringe to the microcannula and retro-injection of the product. Note that the arrows indicate the different directions of product delivery, and it should be noted that the technique does not involve injecting the mucosa area. C-D) Illustration of the same procedures applied to the upper part of the external vulva.

Before the injection, a chlorhexidine aqueous solution is used to perform local asepsis to minimize the risk of infection. Intradermal anesthesia with 2% lidocaine (0.1 mL) is used to create the orifice into which the microcannula will be inserted with a 21-G needle into the subcutaneous tissue. The injection procedure begins with the formation of tunnels in various directions of the subcutaneous plan to cover all regions that need volumization. The HA is retroinjected as the microcannula is being withdrawn, at which point a new tunnel is created to allow the product to cover the entire surface to be treated. Figure 1 depicts a step-by-step breakdown of the technique.

The treated area is gently sculpted and massaged after the injection. The HA used for the present article is the sodium hyaluronate (20 mg/ml) cross-linked with BDDE (1,4-butanediol diglycidyl ether), with a particle size of 900 ± 1100 μm and a minimal free residual BDDE content of ≤2ppm. The amount of HA injected varies depending on the degree of volume deficiency, but 1 mL to 2 mL of the solution of HA is usually sufficient to treat each side. 

All procedures were carried out by an experienced practitioner who had received specialized training in this technique. Patients were closely monitored for any negative effects, such as pain, erythema, or edema, and were instructed to report any complications that occurred during the follow-up period. 

Case description

A 48-year-old woman presented with decreased labia majora volume and associated symptoms. She expressed concerns about her appearance, including deflation and laxity of the labia majora, which led to a loss of confidence. She also had functional issues, such as decreased vaginal lubrication, decreased erotic sensation, and discomfort during intercourse. The patient had no previous history of procedures related to the labia majora and had no relevant or related comorbidities. 

Following a thorough examination of the patient, it was determined that she was a suitable candidate for the procedure. The professional responsible for the procedure also explained all treatment options and the positive and negative aspects of the procedure described in the present article. Given her concerns and the lack of prior interventions or relevant comorbidities, we decided to use our novel technique for restoring volume and tonus to the labia majora using hyaluronic acid (HA) filling. Before starting the procedure, the patient gave informed consent for participation in the article, which included information on the risks and benefits of this article. The procedure included a thorough examination of the patient's labial anatomy and specific concerns. Under local anesthesia, HA was retroinjected into the areas that needed volumization and collagen stimulation using an 18-G, 50-mm-long microcannula. The procedure was carried out with the utmost care, maintaining a sterile environment, ensuring the patient's comfort and safety at all times, keeping open communication with the patient, and providing appropriate pain management to ensure her comfort and safety. 

**Figure 2:**
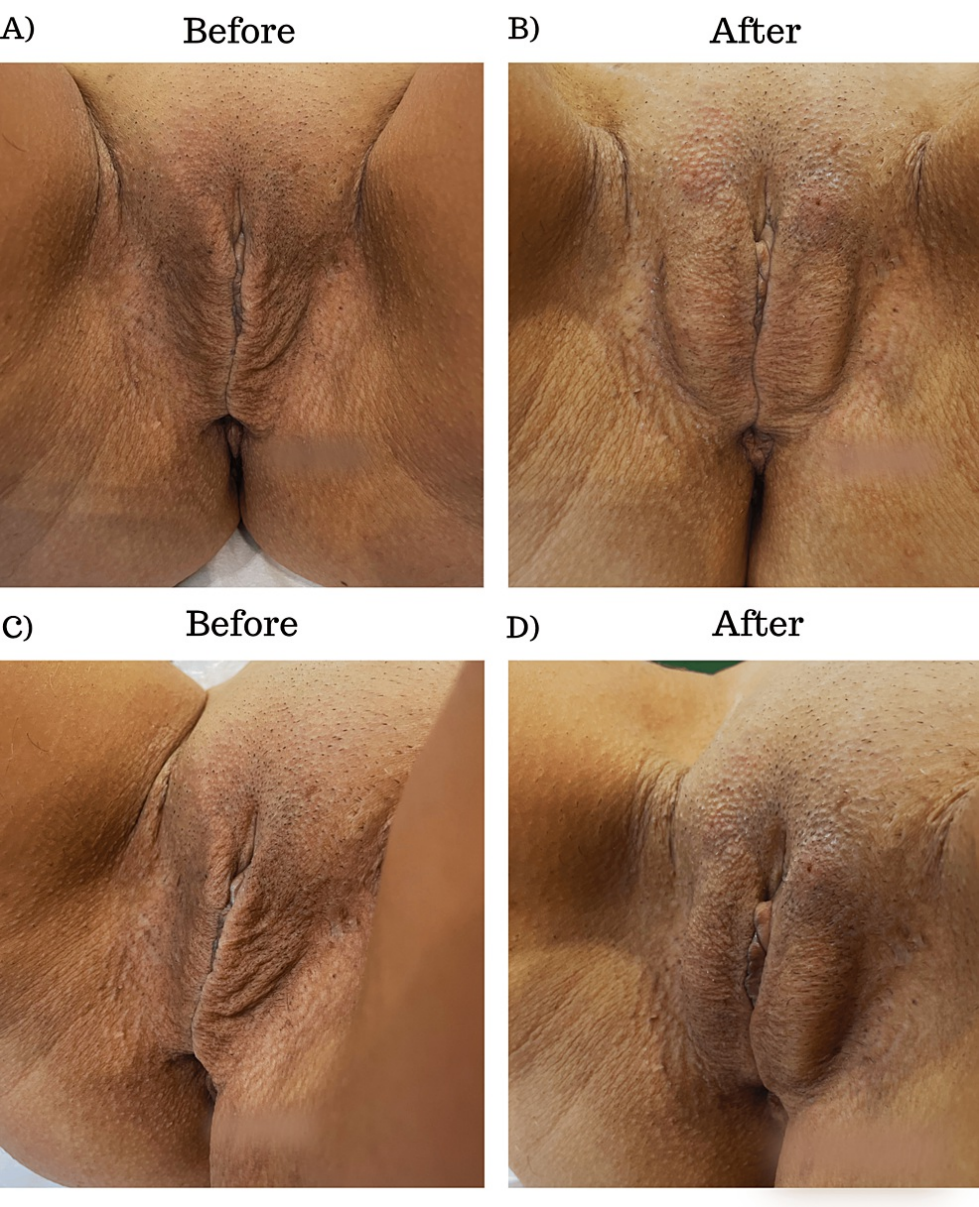
Before and immediately after images of a patient submitted to our procedure. (A) Frontal image of the patient before the procedure. (B) A frontal image of the patient immediately after the procedure. (C) Lateral image of the patient before the procedure. (D) Lateral image of the patient immediately after the procedure.

Immediately after the procedure, noticeable aesthetic improvements were observed, demonstrating the technique's efficacy. The before-and-after images in Figure 2 show the transformation achieved by restoring volume and tonus in the labia majora. The patient's fullness and contour of the treated area improved significantly, giving her a more youthful and rejuvenated appearance. These immediate aesthetic benefits provided a promising outcome, indicating that the hyaluronic acid filling technique was effective in addressing the patient's specific concerns.

The patient had a positive post-procedural experience, reporting no adverse effects such as itching or pain after leaving the clinic. A follow-up appointment was set up to evaluate the patient's progress and any potential side effects or changes in the treated area 12 weeks after the procedure. No significant side effects or complications such as pain, erythema, or edema were observed during the following consultation, suggesting the safety and efficacy of the hyaluronic acid filling technique. Furthermore, during the follow-up, the patient expressed her satisfaction with the procedure's results. She reported increased confidence, a reduction in her aesthetic concerns, and an improvement in her sexual well-being. The patient's high level of satisfaction reinforces the technique's success in achieving the desired results and improving her overall quality of life, aesthetic improvements, and sexual well-being.

## Discussion

External female genitalia clinical anatomy includes the mons pubis, labia majora, labia minora, clitoris, vestibule bulbs, and greater and lesser vestibular glands. It's worth noting that all of these elements have sensory and erectile tissue for sexual arousal. The labia majora is made up of two prominent skin folds that are mostly filled with a finger-like digital process of loose subcutaneous tissue containing smooth muscle, fat tissue, and ligament. It is a structure that protects the clitoris, urethral, and vaginal orifices from friction by enclosing the pudendal cleft. It also aids in the direction of the urinary flow [6-8]. As previously stated in this work, tissue loss is one of the most common complaints from our patients, followed by exposure of clitoris and mucosal tissues, excessive friction during sports practices or in some types of garments, and aesthetic dissatisfaction. Surprisingly, all of these complaints can be explained in part by anatomical changes [9]. 

The re-establishment of volume is immediately noticeable following the procedure. In 15% of all HA injection cases, bruising occurs in the first few days and lasts 3-5 days, whereas swelling occurs in approximately 80% of cases within the first 24 hours after the procedure. We see that the procedure's effects last 12-18 months in our patients. In terms of tissue remodeling, previous studies in arm skin have shown an increase in collagen protein expression that is already visible after four weeks [10]. 

The presented article highlights the successful application of the innovative hyaluronic acid filling technique for the restoration of volume and tonus in the labia majora. The technique described in this study provides a novel and minimally invasive treatment option for women suffering from labia majora atrophy. This technique has the advantage of promoting anatomical, histological, and functional benefits while requiring minimal downtime and no hospitalization. Moreover, this technique offers a non-surgical alternative to procedures like fat transfer, which involve higher risks due to the need for anesthesia during fat collection and the fact that some of the transferred fat may not survive, leading to variable results. 

The procedures described in this article are important for women's mental and physical health because they can alleviate discomfort, promote aesthetic improvements, address medical conditions, and increase body confidence, ultimately promoting overall well-being and a positive sense of self. However, it is important to acknowledge the limitations of this article, including the fact that it presents findings from only one patient, which restricts the ability to assess the effectiveness and generalizability of the results. Additionally, the long-term effects of the technique are not thoroughly evaluated. Furthermore, while several studies have demonstrated certain mechanisms of hyaluronic acid treatment, such as hydration and collagen synthesis, the full extent of these mechanisms remains incompletely understood, particularly with regard to their impact on lymph node structures and function. Therefore, future research should focus on conducting multicenter prospective studies involving a diverse population to provide a more comprehensive understanding of the technique's effectiveness and its mechanisms.

## Conclusions

It is important to note that the technique described in this article is a guideline recommendation, and clinical evaluation of anatomy and functional aspects, as well as technical expertise, are required for the procedure's success and safety. Future research should look into the procedure's long-term effects as well as its potential impact on sexual function and quality of life. Overall, this procedure is a safe and effective option for women who want to improve both the functional and aesthetic aspects of their genitalia.
